# EHMTI-0043. Clinically-oriented stratified approach and patient-preference approach in the management of MOH – a comparison between two strategies

**DOI:** 10.1186/1129-2377-15-S1-C56

**Published:** 2014-09-18

**Authors:** P Rossi, J Faroni, C Tassorelli

**Affiliations:** 1 Headache Centre, INI, Grottaferrata (Rome), Italy; 2 Headache Science Centre, C. Mondino Foundation, Pavia, Italy

## Background and aim

The management of medication overuse headache (MOH) is often difficult and no specific guidelines are available as regards the most practical and effective approaches. The aim of this study is to compare the cost-effectiveness of two different stratified approaches of drug withdrawal in 100 MOH subjects.

## Materials and methods

In the first approach, called clinically-oriented stratified strategy (COSS), patients were stratified based on the presence /absence of complicated MOH. Patients with complicated MOH received a standard inpatient withdrawal programme whereas patients with simple MOH received an advice to withdraw the offending drug. In the second approach, called patient-preference stratified strategy (PPSS), the patients received information about the effectiveness of the different withdrawal regimens (1) simple advice, 2) outpatient detoxification and 3) inpatient detoxification) and they were treated according to their preferences.

The primary outcome measures used for comparing COSS and PPSS were: 1) number of responders ; 2) number of dropouts 3) cost per detoxified patient, 4) number of relapsers at 6 months follow-up.

## Results

The Number of responders (80% vs 70%) , dropouts (16% vs 18%) and relapsers (12.5 vs 14.5%) did not differ between COSS and PPSS. The cost per detoxified patient was 1211 € for COSS and 430 € for PPSS.

## Conclusions

A patient preference stratified strategy is as effective as a clinically-oriented stratified strategy, but less cost intense.

No conflict of interest.

